# Prevalence and factors associated with HIV treatment non-adherence among people living with HIV in three regions of Cameroon: A cross-sectional study

**DOI:** 10.1371/journal.pone.0283991

**Published:** 2023-04-04

**Authors:** Amos Buh, Raywat Deonandan, James Gomes, Alison Krentel, Olanrewaju Oladimeji, Sanni Yaya

**Affiliations:** 1 Interdisciplinary School of Health Sciences, University of Ottawa, Ottawa, Ontario, Canada; 2 School of Epidemiology and Public Health, University of Ottawa, Ottawa, Ontario, Canada; 3 Department of Public Health, Faculty of Health Sciences, Walter Sisulu University, Mthatha, Eastern Cape, South Africa; 4 Faculty of Health Sciences, Durban University of Technology, Durban, South Africa; 5 School of International Development and Global Studies, University of Ottawa, Ottawa, Ontario, Canada; 6 The George Institute for Global Health, Imperial College London, London, United Kingdom; University of Botswana School of Medicine, BOTSWANA

## Abstract

**Background:**

In Cameroon, HIV care decentralization is enforced as a national policy, but follow-up of people living with HIV (PLWH) is provider-driven, with little patient education and limited patient participation in clinical surveillance. These types of services can result in low antiretroviral therapy (ART) adherence. The objective of this study was to assess the prevalence and predictors of ART non-adherence among PLWH in Cameroon.

**Methods:**

A cross-sectional descriptive study of PLWH in HIV treatment centres in Cameroon was conducted. Only PLWH, receiving treatment in a treatment centre within the country, who had been on treatment for at least six months and who were at least 21 years old were included in the study. Individuals were interviewed about their demographics and ART experiences. Data were collected using a structured interviewer-administered questionnaire and analyzed using STATA version 14.

**Results:**

A total of 451 participants participated in this study, 33.48% were from the country’s Southwest region. Their mean age was 43.42 years (SD: 10.42), majority (68.89%) were females. Overall proportion of ART non-adherence among participants was 37.78%, 35.88% missed taking ART twice in the last month. Reasons for missing ART include forgetfulness, business and traveling without drugs. Over half of participants (54.67%) know ART is life-long, 53.88% have missed ART service appointments, 7.32% disbelieve in ART benefits, 28.60% think taking ART gives unwanted HIV Status reminder and 2.00% experienced discrimination seeking ART services. In the multivariate analysis, odds of ART non-adherence in participants aged 41 and above was 0.35 times (95%CI: 0.14, 0.85) that in participants aged 21–30 years, odds of ART non-adherence comparing participants who attained only primary education to those who attained higher than secondary education was 0.57 times (95%CI: 0.33, 0.97) and the odds of ART non-adherence in participants who are nonalcohol consumers was 0.62 times (95%CI: 0.39, 0.98) that in alcohol consumers.

**Conclusion:**

High proportion of participants are ART non-adherent, and the factors significantly associated with ART non-adherence include age, education and alcohol consumption. However, some reasons for missing ART are masked in participants’ limited knowledge in taking ART, disbelief in ART benefits, feelings that ART gives unwanted HIV status reminder and experiencing discrimination when seeking ART services. These underscores need to improve staff (health personnel) attitudes, staff-patient-communication, and proper ART prior initiation counselling of patients. Future studies need to focus on assessing long-term ART non-adherence trends and predictors using larger samples in many treatment centres and regions.

## Background

The HIV/AIDS pandemic remains a major global public health problem. From the start of the epidemic to the end of 2021, 84.2 million (64.0–113.0 million) people have been infected with HIV and about 40.1 million (33.6–48.6 million) people have died of HIV [1]. In 2021, an estimated 38.4 million people were living with HIV worldwide, 1.5 million new infections were registered and 650,000 (510,000–860,000) deaths from AIDS-related illnesses were reported [1, 2]. Despite efforts to prevent new infections and deaths, sub-Saharan Africa (SSA) or the World Health Organization (WHO) African region, bears the heaviest burden of HIV with nearly 1 in every 25 adults (3.4%) living with HIV and accounting for more than two-thirds of people living with HIV worldwide [1, 3]. Cameroon is one of the countries with a high prevalence of HIV in the West and Central African Sub Region [4]. The prevalence of HIV in this country stands at 2.9% [5, 6]. In 2021, 15000 new cases of the disease were recorded but only 78% of people living with HIV (PLWH) are on antiretroviral therapy in Cameroon [6–8].

Evidence informs that once people are diagnosed as HIV-positive, it is important to ensure that they can be effectively linked to care [9, 10] and since no cure has been discovered yet, the only modality of treatment to prolong life and to improve the quality of life of people living with HIV/AIDS at present is anti-retroviral therapy (ART) [10–12]. It has also been documented that ART regimens are life-long requirement of strict compliance by patients and needs to be taken as prescribed to achieve treatment success and prevent drug resistance [13–15]. Therefore, to be effective, ART has to be taken life-long with 100% adherence, as clinical and immunological improvement as well as viral suppression are only expected when individuals adhere to ART [10, 13, 14, 16]. Poor or non-adherence–not taking ART every day and exactly as prescribed, can lead to drug resistance and treatment failure [10, 17].

However, non-adherence to ART is still observed in SSA countries where the burden of the disease is high [18]. Some studies have reported that the patient, disease, therapy and relationship of patients with healthcare providers are associated with ART adherence issues [19, 20]. Nevertheless, factors affecting non-adherence to HIV treatment are not well known especially in regions with insecurity as well as in a resource poor setting like Cameroon.

In Cameroon, decentralization of HIV care has been enforced as a national policy, with few attempts to examine the performance of this policy and its role in HIV prevention [21]. Apart from that, the follow-up of PLWH in Cameroon is provider oriented with little patient education and limited patient involvement in clinical surveillance [22]. Little is known about the proportion of PLWH who do not adhere to this kind of treatment in the country.

Furthermore, the healthcare system in Cameroon often experiences low access to drugs, inadequate staffing, coordination and management of commodities for HIV. A few studies have been conducted, all in the country’s capital city (Yaoundé), to assess patient adherence to ART and examine the socio-economic, clinical and psychological factors associated with self-reported ART interruption [18, 23, 24]. There is limited or no literature on the socio-demographic/structural factors associated with non-adherence to HIV treatment in this country. The aim of this study therefore was to determine the prevalence and predictors of HIV treatment non-adherence among PLWH in the Northwest, Southwest and Littoral regions of Cameroon.

## Methods

### Study design and setting

A cross-sectional descriptive study was conducted on adult PLWH who were receiving ART treatment in selected HIV treatment centres in three regions of Cameroon.

Briefly, the study was conducted in Cameroon–a country in sub-Saharan Africa with a high prevalence of HIV. The country has a population of over 28 million inhabitants [25], it is divided into ten regions and shares boundaries with Nigeria in the west, Chad in the north, Central African Republic in the east and Gabon, Equatorial Guinee, Congo in the south. Participants for the study were enrolled in HIV treatment centres in three selected regions (Littoral, Southwest and Northwest regions–all selected through balloting) of the country. In each of the selected regions, the study was conducted in only one HIV treatment centre (Mboppi Baptist Hospital Douala in the Littoral region, the Regional Hospital Buea in the Southwest region, and the Nkwen Baptist Hospital Bamenda in the Northwest region) that was purposefully selected based on probability proportionate to the size of patients registered in the centre. The centres were chosen such that on average, about 20% of PLWH and registered for ART in the centres were recruited per week in order to achieve the required sample size in a reasonable short period of time. In all the selected treatment centres, patients are managed and followed-up by doctors, nurses, and other health personnel. Patients are received in the centres from Monday to Friday, between 8:00 a.m to 3:00 p.m. Each centre has a reception room where patients are registered in order of their arrival, a waiting room where patients wait before being consulted and consultation rooms where patients are consulted and counseled.

### Study population, participants, and sampling

The study targeted PLWH who were receiving ART in HIV treatment centres in the Northwest, Southwest and Littoral regions of the country. To be eligible for the study, a participant had to be a patient living with HIV, receiving treatment, or being followed up in a treatment centre within the country, be at least 21 years old, had been on treatment for at least six months and must have consented to participate in the study.

The sample size for the study was calculated using the formula for estimating the sample size of a single population proportion for a prevalence study of an infinite population [26]. Since the proportion of patients who do not adhere to ART in Cameroon was not known, we assumed the prevalence of non-adherence to ART in Cameroon was 50%; and with a margin of error of 5% at 95% confidence level, a sample size of 384 was determined. However, we anticipated having a non-response rate of 10% from participants in the study. To account for this, we increased the 384-sample by 10% to have a sample size of 422. Nonetheless, in order to have a large enough sample that will enable us to estimate the prevalence of non-adherence to ART in our study we enrolled 451 participants in this study.

Participants were sampled and enrolled into the study between the period of November and December 2021 by six (6) trained interviewers (two from each of the three selected regions where the study was conducted) with a background training in health. A multistage sampling technique was used to select participants for this study. Firstly, a list of all the ten regions of the country with HIV treatment centres and their corresponding populations of PLWH was made. Next, balloting was used to select three regions and in each selected region, the HIV treatment centre with a high population of PLWH registered was further selected. The overall sample size was divided into three to obtain the number of participants to interview in each centre. Study interviewers used the systematic sampling approach in each centre to recruit participants. After providing the usual care in each centre, clinicians referred selected patients to study interviewers who then explained the study to each eligible patient.

### Data collection

Data on demographic/structural characteristics of participants and participants’ experiences taking ART was collected using a standardized ART adherence self-reported questionnaire (S1 File) adapted from similar studies [27–31]. Prior to data collection, the questionnaire was pretested in an ART treatment centre (not included in the study) in Cameroon. In this study, we considered participants non-adherent to ART to be those participants who reported having once missed treatment (missed taking their ART in the past 4 weeks prior to participating in the study at least one time). Our data collection was monitored weekly in each centre and collected data was entered in an electronic questionnaire for further analysis.

### Data management and analysis

The collected data was entered into an MS Access interface on Epi-info and analyzed using the statistical software program STATA version 14. The participants’ socio-demographic/structural characteristics have been described using frequencies and percentages for categorical variables and means, medians, inter-quartile ranges and standard deviations for continuous variables.

To determine the proportion of participants who have not adhered to HIV treatment, the percentage of participants who reported having once missed treatment was computed.

To investigate the socio-demographic/structural factors associated with non-adherence to HIV treatment, bivariate and multivariate analysis were done. The bivariate analysis comprised of using non-adherence to HIV treatment as a binary outcome variable and participants’ socio-demographic/structural characteristics as predictors. The odds of not adhering to ART between participants using unadjusted odds ratios, 95% confidence intervals and P-values were computed. A P-value ≤ 0.25 was set as the determining point in the bivariate analysis for a variable to be considered as appearing to have an association with non-adherence to HIV treatment and be included in the multivariate logistic model [32]. For the multivariate analysis, non-adherence to HIV treatment was considered as a binary outcome variable and all variables with P-values ≤ 0.25 in the bivariate analysis were considered as predictors and included in the multivariate logistic regression model. Adjusted odds ratios, 95% confidence intervals and p-values were computed. All variables with p-values < 0.05 were considered to have a statistically significant association with non-adherence to HIV treatment (ART).

#### Ethics approval and consent to participate

Ethical clearance for the study was obtained from the Health Sciences and Science Research Ethics Board (REB) of the University of Ottawa (Ethics File Number: H-08-21-7274) and the Cameroon Baptist Convention Health Board Institutional Review Board (CBCHBIRB) (IRB study number: IRB2021-53). Administrative authorization was also obtained from the Regional Delegate of Public Health of the Southwest Region (Ref: 1211/MINSANTE/SWR/RDPH/P5/810/725) and the Regional Hospital Buea (Ref /MPH/SWRDPH/BRH/IRB). Written informed consent was obtained from all participants prior to their participation in the study.

## Results

### Participants’ characteristics

The characteristics of the 451 participants included in this analysis are presented in Table 1. More than one third of the participants in the study 151 (33.48%) were from the Southwest region of the country. The mean age of participants was 43.42 years (SD: 10.42) and majority of the participants (68.89%) were females. Two hundred and one (44.57%) of the participants were married and 156 (34.59%) had attained only the primary school level of education. One hundred and eighty-three (40.58%) of participants were Roman Catholic Christians. Two hundred and six (45.78%) of the participants were self-employed and the median monthly income of participants was 50,000 XAF (IQR: 30,000–80,000XAF). Both the median number of months of participants since they were first diagnosed HIV positive, and the median number of months participants have been taking ART was 84 months (IQR: 48–132). A vast majority of the participants 402 (89.33%) had a once daily ART dosing frequency. Most participants’ 344 (76.44%) means of transportation from their homes to the HIV treatment centre was by car/taxi, with the median distance from participants homes to the HIV treatment centre being 8 Km (IQR:5–17) and the median time spent by participants from their homes to the treatment centre being 30 minutes (IQR: 15–45). Participants had a mean number of 5 people (SD: 2.62%) living in their homes with 42.54% of those people being their children and majority of the participants 363 (80.85%) had disclosed their HIV status to their family/friends.

**Table 1:** Characteristics of the overall study population (N (%) or Mean (SD) or Median (IQR)).

Characteristic	N or Mean or Median	% or SD or IQR
Region of residence		
Northwest	150	33.26
Southwest	151	33.48
Littoral	150	33.26
Age (years)	43.42	10.42
Sex		
Male	140	31.11
Female	310	68.89
Marital status		
Single	170	37.69
Married	201	44.57
Divorced	17	3.77
Separated	18	3.99
Never married	19	4.21
Widowed/widower	26	5.76
Education*		
Primary	156	34.59
Secondary	153	33.92
High school	73	16.19
University	69	15.30
Religion		
Muslim	17	3.77
Catholic	183	40.58
Protestant	169	37.47
Pentecostal	79	17.52
Animist	3	0.67
Working status		
No job	138	30.67
Self-employed	206	45.78
Government staff	49	10.89
Private staff	57	12.67
Monthly income (XAF)	50000	30000–80000
Distance to centre (KM)	8	5–17
Time to centre (minutes)	30	15–45
Transportation means to centre		
By foot	43	9.56
Bike	63	14.00
Car/taxi	344	76.44
Number of people in home	5	2.62
Persons living with		
Spouse/partner	20	4.45
Children	191	42.54
Parents	29	6.46
Relatives	178	39.64
Nobody	31	6.90
Disclosed HIV status to family/friend		
No	86	19.15
Yes	363	80.85
HIV months	84	48–132
ART months	84	48–132
ART daily dosing		
Once daily	402	89.33
Twice daily	48	10.67

*Primary education involves at most seven years, secondary at most twelve, high school at most fourteen and university above fourteen years of education. N = frequency, % = frequency in percentage, SD = standard deviation and IQR = inter quartile range, KM = Kilometers, XAF = African Financial Community Franc.

### Non-adherence to ART among participants

**Fig 1:**
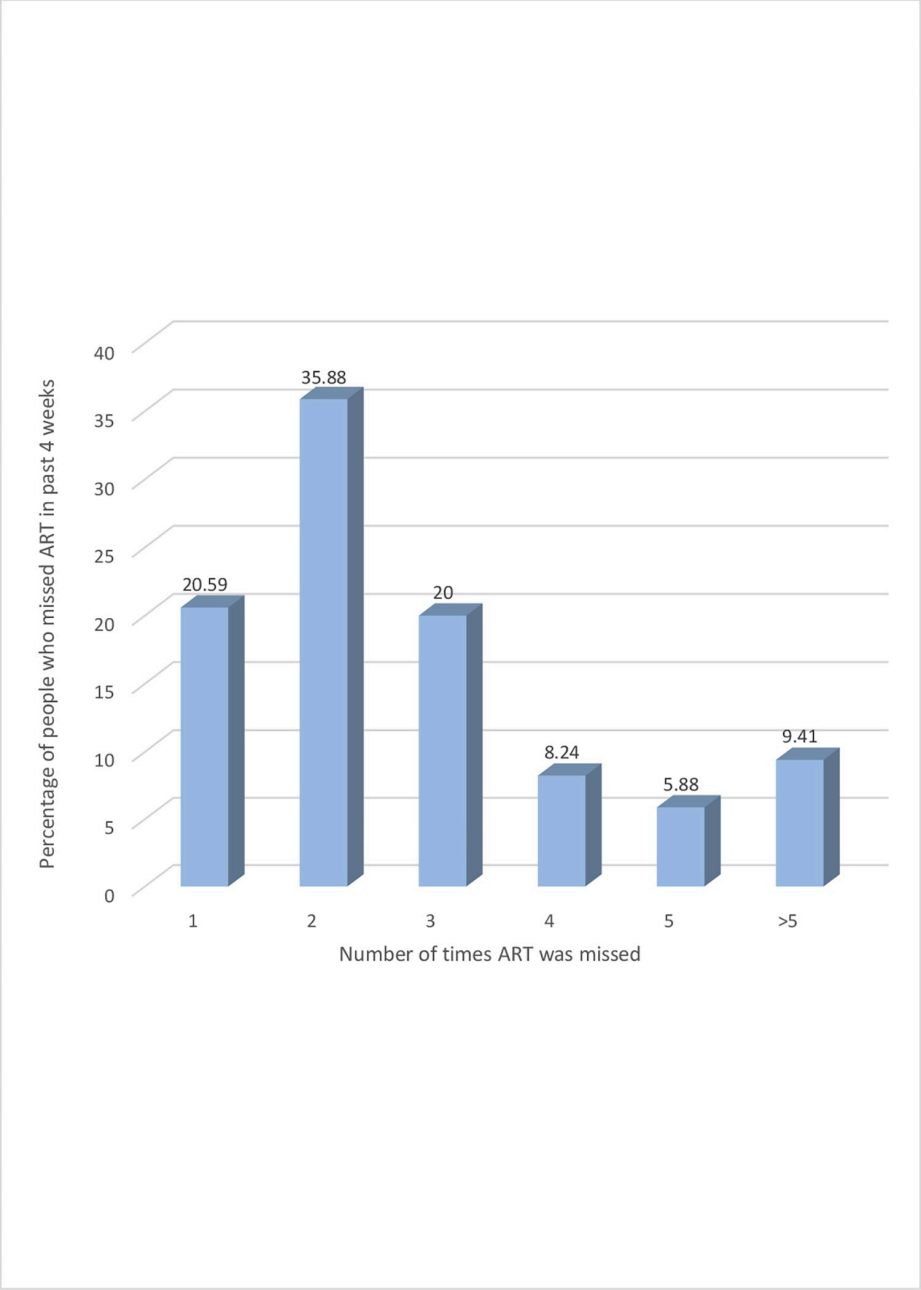
Proportion of patients who missed ART in past 4 weeks versus number of times ART was missed.

**Fig 2:**
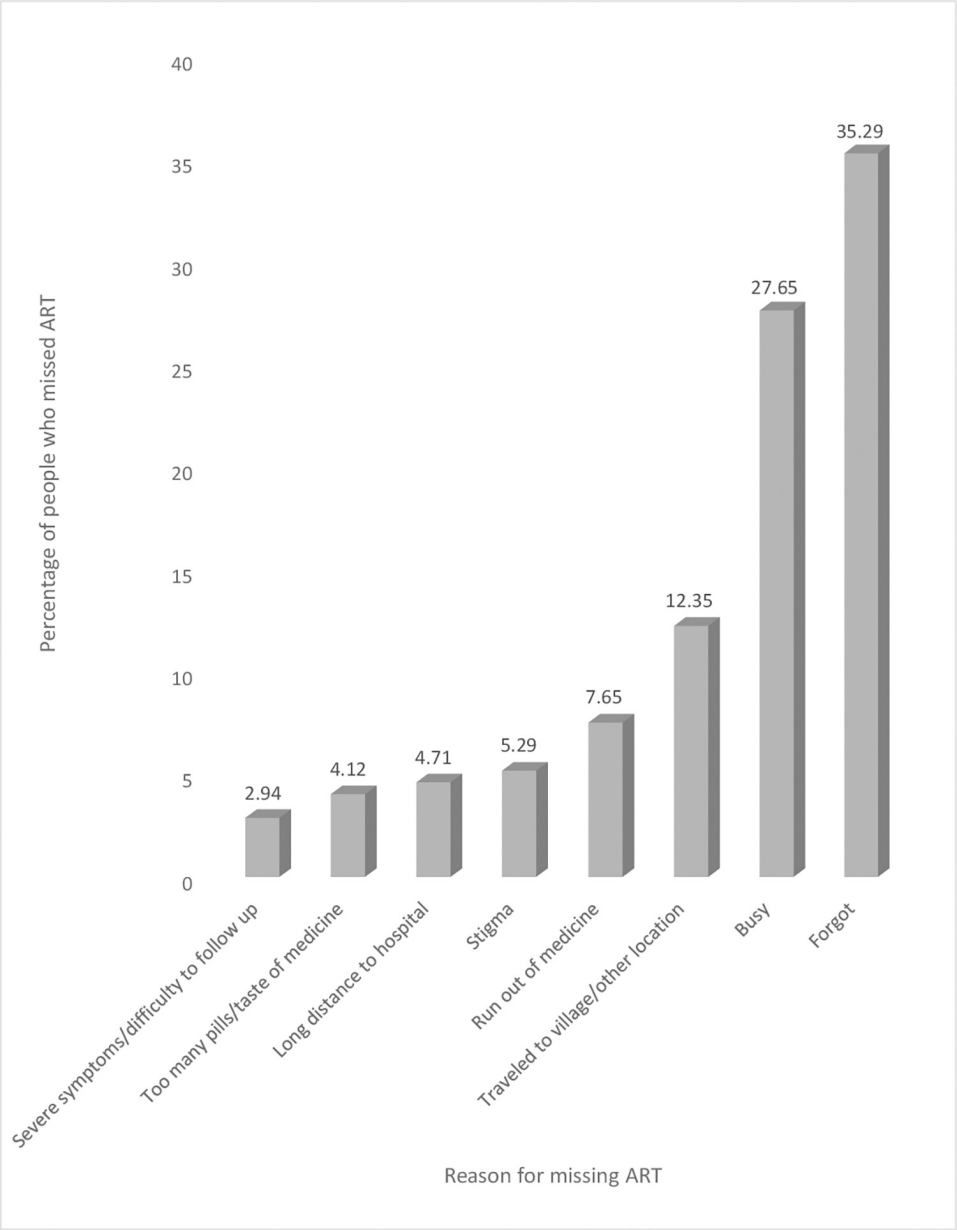
Proportion of patients that missed ART versus reasons ART was missed.

Table 2, Figs 1 and 2 shows participants’ ART non-adherence characteristics. Overall, the proportion of participants that did not adhere to their ART (defined as ever missed taking ART at least once in the last month prior to their participation in this study) was 37.78% (Table 2). The number of times participants missed taking their ART in the last four weeks varied from once to more than five times–with more than one third of these participants 61 (35.88%) missing their ART twice (Fig 1).

**Table 2:** ART adherence, health risk behaviors and HIV related hospitalizations among participants.

Variable	N	%
**Ever missed taking ART last month**		
No	280	62.22
Yes	170	37.78
**HIV related hospitalization**		
**No**	327	72.67
Yes	123	27.33
**Reasons for hospitalization**		
**Chronic diarrhea**	14	11.29
Chronic herpes simplex	7	5.65
TB/pneumocystis	15	12.10
Meningitis	4	3.23
Prolong fever	53	42.74
Weight loss	9	7.26
Jaundice	2	1.61
Other (malaria, anemia, cancer, diabetes, toxoplasmosis, candidiasis)	20	16.13
**Health risk behaviors**		
Smoking	18	3.99
Alcohol	164	36.36
Unprescribed drug use	113	25.17
Multiple sex partners	39	8.67
No physical exercises	261	58.00

N = frequency, % = frequency in percentage

With regards to the reasons for missing their ART in the last four weeks, 60 (35.29%) of the participants stated that they forgot, 27.65% of the participants stated that they were busy while 12.35%, 7.65% and 5.29% of the participants respectively stated traveled to the village/other location, run out of medicines and stigma as their reasons for missing their ART (Fig 2).

### HIV related hospitalizations among participants

The proportion of participants that had been hospitalized for HIV-related concerns prior to their participation in this study was 27.33%. Prominent among the specific reasons for hospitalization was prolonged fever—reported by 42.74% of participants. However, TB/pneumocystis, chronic diarrhea, and other health issues (malaria, anemia, cancer, diabetes, toxoplasmosis, candidiasis) were respectively reported in 12.10%, 11.29% and 16.13% of patients (Table 2).

### Health risk behaviors among participants

The health risk behaviors practiced by participants are presented in Table 2. A proportion of participants were engaged in health risky behaviors such as consumption of alcohol and no physical activity—reported in 36.36% and 58.00% of the participants respectively. Also, use of unprescribed drugs was reported in 25.17% of participants as well as having multiple sex partners which was reported in 8.67% of participants.

### Participants’ knowledge, experiences, and views about ART

The views and experiences of participants are presented in Table 3. Over half (54.67%) of the participants know and strongly agree that ART is lifelong, with 257 (57.11%) of the participants reporting that their health status has been stable since ART initiation. Nevertheless, 243 (53.88%) of participants reported having ever missed an ART service appointment. Although 279 (61.86%) of participants disagreed that they experienced physical adverse events related to ART, 84 (18.67%) of participants reported having ever experienced ARV side effects, 33 (7.32%) of participants do not believe in the benefits of ART and 129 (28.60%) of participants strongly agree that taking ART tablets gives them an unwanted reminder of their HIV status. One hundred and seventy-six (39.02%) of the participants did not express any worries that ART might stop working in future, but a small proportion of the participants (2.00%) reported that they had experienced health service discrimination while seeking HIV treatment services.

**Table 3:** Participants’ knowledge, experiences and views about ART.

Variable	N	%
**Health status since ART initiation**		
Worse	4	0.89
Stable	257	57.11
Better	189	42.00
**Ever had ARV side effects**		
No	366	81.33
Yes	84	18.67
**Ever missed ART service appointment**		
No	208	46.12
Yes	243	53.88
**Know ART is life long**		
**Strongly agree**	246	54.67
Agree	157	34.89
Uncertain	24	5.33
Disagree	12	2.67
Strongly disagree	11	2.44
**Do not believe in benefits of ART**		
**Strongly agree**	26	5.76
Agree	33	7.32
Uncertain	48	10.64
Disagree	209	46.34
Strongly disagree	135	29.93
**Taking tablets gives unwanted HIV status reminder**		
**Strongly agree**	129	28.60
Agree	106	23.50
Uncertain	38	8.43
Disagree	131	29.05
Strongly disagree	47	10.42
**Worried ART will stop working in future**		
**Strongly agree**	37	8.20
Agree	54	11.97
Uncertain	123	27.27
Disagree	176	39.02
Strongly disagree	61	13.53
**Experienced physical adverse events in last 12 months**		
**Strongly agree**	18	3.99
Agree	37	8.20
Uncertain	10	2.22
Disagree	279	61.86
Strongly disagree	107	23.73
**Experienced health service discrimination in last 2 years**		
**Strongly agree**	9	2.00
Agree	4	0.89
Uncertain	9	2.00
Disagree	167	37.03
Strongly disagree	262	58.09

N = frequency, % = frequency in percentage

### Correlates of participants’ non-adherence to ART

The sociodemographic/structural correlates of patients’ non-adherence to ART offered in treatment centres in Cameroon are presented in Tables 4 and 5. In the bivariable analysis, the factors that appeared to be associated with patients’ non-adherence to ART included sex (being female when compared to being male), being of age 31–40 and 41 and above when compared to age 21–30, being married/cohabiting and being divorced/separated/widow when compared to being single/never married, attaining primary and secondary level of education when compared to those that attained higher than secondary level of education, being 5–9 years and 10 or more years since HIV positive diagnosis when compared to being less than one year since diagnosis, taking ART for 5–9 years and 10 or more years when compared to taking ART for less than one year, having no job when compared to having a self-employed/ government/ private sector job, being a Muslim/Animist/Pentecostal Christian when compared to being a protestant Christian, having more than two people living in their homes when compared to having two or less people living in their homes, living with their children when compared to living with their parents, being a nonsmoker when compared to being a smoker and being a nonalcoholic when compared to being an alcoholic. In fact, the odds of non-adherence to ART in female participants was 0.77 times (95%CI: 0.51, 1.16) that in male participants. The odds of non-adherence to ART comparing participants aged 41 and above to participants 21–30 years of age was 0.34 times (95%CI: 0.18,0.64). Also, the odds of non-adherence to ART in divorced/separated/widowed participants was 0.60 times (95%CI: 0.33, 1.11) that in single/never married participants. The odds of not adhering to ART in participants with secondary or less level of education was 0.66 times (95%CI: 0.42, 1.06) that in participants that had attained greater level of education beyond secondary school. With regards to the number of years since HIV positive diagnosis, the odds of not adhering to ART in participants who were 10 or more years since their HIV positive diagnosis was 0.35 times (95%CI: 0.14, 0.91) that in participants who had not been up to a year since their HIV positive diagnosis. Again, the odds of not adhering to ART in participants 10 or more years since their initiation of ART was 0.37 times (95%CI: 0.14, 0.98) that in participants who had been on ART for less than a year. The odds of not adhering to ART comparing participants who had no job to participants who were either self-employed or working with the government or private sector was 0.76 times (95%CI: 0.50, 1.16). when considering participants’ religion, the odds of not adhering to ART in participants who were either Muslim, Animist or Pentecostal in faith was 0.67 times (95%CI: 0.40,1.13) that in participants who were protestant Christians. The odds of not adhering to ART comparing participants living with more than 2 persons in their homes to those living with 2 or lesser persons was 0.75 times (95%CI: 0.47, 1.19) while the odds of not adhering to ART in participants living with their children was 0.43 times (95%CI: 0.20, 0.95) that in participants living with their parents. When comparing nonsmokers to smokers, the odds of not adhering to ART was 0.47 times (95%CI: 0.18, 1.22). Finally, the odds of not adhering to ART in participants who said they do not drink alcohol was 0.59 times (95%CI: 0.40, 0.87) that in participants who said they drink alcohol (Table 4).

**Table 4:** Correlates of participants non-adherence with ART- bivariable analysis.

Characteristic	N	%	Participants non-adherence with ART		P-values
			OR*	95% CI	
Region of residence					
Southwest	59	39.07	Ref		
Northwest	55	36.91	0.91	0.57–1.45	0.70
Littoral	56	37.33	0.93	0.58–1.48	0.76
Age					
21–30	29	59.18	Ref		
31–40	50	39.68	0.45	0.23–0.89	**0.02**
41+	91	33.09	0.34	0.18–0.64	**<0.01**
Sex					
Male	59	42.14	Ref		
Female	111	35.92	0.77	0.51–1.16	**0.21**
Marital status					
Single/never married	81	42.86	Ref		
Married /cohabiting	70	35.00	0.72	0.48–1.08	**0.11**
Divorced/separated/widow	19	31.15	0.60	0.33–1.11	**0.11**
Education**					
Primary	48	30.97	0.52	0.32–0.83	**0.01**
Secondary	56	36.60	0.66	0.42–1.06	**0.09**
Greater than secondary	66	46.48	Ref		
HIVyrs***					
<1	11	55.00	Ref		
1–4	53	50.00	0.82	0.31–2.14	0.68
5–9	55	35.48	0.45	0.18–1.15	**0.10**
10+	51	30.18	0.35	0.14–0.91	**0.03**
ARTyears					
<1	10	52.63	Ref		
1–4	53	49.53	0.88	0.33–2.35	0.80
5–9	60	36.59	0.52	0.20–1.35	**0.18**
10+	47	29.38	0.37	0.14–0.98	**0.05**
Working status					
Self-employed/ government/ private staff	123	39.55	Ref		
No job	46	33.33	0.76	0.50–1.16	**0.21**
Distance					
≤25	146	37.63	0.96	0.55–1.66	0.87
>25	24	38.71	Ref		
Religion					
Protestant	68	40.48	Ref		
Catholic	71	38.80	0.93	0.61–1.43	0.75
Muslim/ Animist/ Pentecostal	31	31.31	0.67	0.40–1.13	**0.14**
Monthly income (XAF)					
≤50000	74	39.57	0.85	0.46–1.55	0.59
51000–100000	29	36.25	0.73	0.36–1.48	0.39
>100000	24	43.64	Ref		
Transportation means to centre					
Bike	27	42.86	Ref		
Car/taxi	127	37.03	0.78	0.45–1.35	0.38
By foot	16	37.21	0.79	0.36–1.75	0.56
Number of people in home					
≤2	42	43.30	Ref		
>2	121	36.45	0.75	0.47–1.19	**0.22**
Persons living with					
Parents	15	51.72	Ref		
Children	60	31.58	0.43	0.20–0.95	**0.04**
nobody	13	41.94	0.67	0.24–1.87	0.45
Relatives	72	40.45	0.63	0.29–1.39	0.26
Spouse/partner	10	50.00	0.93	0.30–2.92	0.91
Smoke					
Yes	10	55.56	Ref		
No	160	37.04	0.47	0.18–1.22	**0.12**
Alcohol					
Yes	75	45.73	Ref		
No	95	33.22	0.59	0.40–0.87	**0.01**
Unprescribed drugs					
Yes	45	39.82	Ref		
No	125	37.31	0.90	0.58–1.39	0.64

*OR = unadjusted odds ratio

**Primary education at most seven years, secondary education is at most five years after primary education and above five years of education after primary education for greater than secondary

***HIVyrs = number of years since HIV-positive diagnosis, OR = odds ratio, Ref = reference variable category, CI = confidence interval, P-values<0.25 suggests possible association to satisfaction, XAF = African Financial Community Franc.

**Table 5:** Correlates of participants non-adherence with ART- multivariable analysis.

Characteristic	N	%	Participants non-adherence with ART		P-values
			aOR*	95% CI	
Age					
21–30	29	59.18	Ref		
31–40	50	39.68	0.42	0.17–1.02	**0.05**
41+	91	33.09	0.35	0.14–0.85	**0.02**
Sex					
Male	59	42.14	Ref		
Female	111	35.92	1.04	0.64–1.70	0.87
Marital status					
Single/never married	81	42.86	Ref		
Married /cohabiting	70	35.00	0.95	0.55–1.61	0.84
Divorced/separated/widow	19	31.15	0.84	0.40–1.74	0.63
Education**					
Primary	48	30.97	0.57	0.33–0.97	**0.04**
Secondary	56	36.60	0.66	0.40–1.10	0.11
Greater than secondary	66	46.48	Ref		
HIVyrs***					
<1	11	55.00	Ref		
1–4	53	50.00	1.22	0.09–16.38	0.88
5–9	55	35.48	0.12	0.01–2.57	0.18
10+	51	30.18	0.23	0.01–5.64	0.37
ARTyears					
<1	10	52.63	Ref		
1–4	53	49.53	0.78	0.05–11.14	0.85
5–9	60	36.59	5.26	0.22–124.78	0.30
10+	47	29.38	2.00	0.07–55.17	0.68
Working status					
Self-employed/ government/ private staff	123	39.55	Ref		
No job	46	33.33	0.66	0.39–1.10	0.11
Religion					
Protestant	68	40.48	Ref		
Catholic	71	38.80	0.83	0.51–1.34	0.45
Muslim/ Animist/ Pentecostal	31	31.31	0.78	0.43–1.40	0.40
Number of people in home					
≤2	42	43.30	Ref		
>2	121	36.45	1.04	0.57–1.89	0.89
Persons living with					
Parents	15	51.72	Ref		
Children	60	31.58	1.01	0.38–2.70	0.98
nobody	13	41.94	1.55	0.33–7.16	0.58
Relatives	72	40.45	1.34	0.52–3.46	0.54
Spouse/partner	10	50.00	1.82	0.49–6.80	0.37
Smoke					
Yes	10	55.56	Ref		
No	160	37.04	0.45	0.13–1.54	0.20
Alcohol					
Yes	75	45.73	Ref		
No	95	33.22	0.62	0.39–0.98	**0.04**

*aOR = adjusted odds ratio

**Primary education at most seven years, secondary education is at most five years after primary eduction and above five years of education after primary education for greater than secondary

***HIVyrs = number of years since HIV-positive diagnosis, OR = odds ratio, Ref = reference variable category, CI = confidence interval, P-values<0.05 are statistically significant.

After adjusting for potential confounding by each of the socio-demographic/ structural characteristics that appeared to have an association with non-adherence to ART in the bivariate analysis, only being of a younger age (21–30), attaining only the primary level of education and being an alcohol consumer remained significant predictors of non-adherence to ART. The odds of not adhering to ART in participants aged 41 and above was 0.35 times (95%CI: 0.14, 0.85) that in participants aged 21–30 years. Similarly, the odds of not adhering to ART in participants aged 31–40 was 0.42 times (95%CI: 0.17, 1.02) that in participants who were aged 21–30 years. Also, the odds of not adhering to ART comparing participants who attained only a primary level of education to those who attained a level of education higher than the secondary school was 0.57 times (95%CI: 0.33, 0.97). Finally, the odds of not adhering to ART in participants who said they do not consume alcohol was 0.62 times (95%CI: 0.39, 0.98) that in participants who said they were alcohol consumers (Table 5).

## Discussion

The only treatment modality to prolong life and improve quality of life in people living with HIV/AIDS (PLWHA) is anti-retroviral therapy (ART) [17, 33]. ART has completely revolutionized the course of the HIV disease, transforming the HIV infection from a life-threatening infection to a manageable chronic disease [27]. It prevents further replication or multiplication of the virus, reduces the patient’s viral load, increases CD4 counts, reduces the likelihood of opportunistic infections and patient hospitalizations, improves patient’s quality of life and reduces mortality [11, 12, 34]. Adherence to ART reduces the viral load in an individual’s body, prevents treatment failure and the likelihood of emergence of drug-resistant viral strains, and also prevents further transmission of the virus to uninfected individuals [17, 35–37].

In this study assessing the extent of ART non-adherence among PLWH treated in HIV treatment centres in the Northwest, Southwest and Littoral regions of Cameroon, we evaluated the proportion of PLWH who are ART non-adherent, the number of times ART was missed in the last month and reasons for non-adherence to treatment. We document that the proportion of ART non-adherence in patients in these centres is high. Approximately, 38% of patients are non-adherent to their ART, a high proportion of concern since ART must be taken as prescribed for life, with 100% adherence for it to be effective [13, 16, 33]. Also, the frequency with which participants missed their ART (between one and more than five times in the past month) and reasons for missing ART (such as forgetfulness, preoccupied with other things, traveling somewhere else without drugs) are also documented. In this study, the available socio-demographic/structural characteristics of the participants, both individually and as a group, did not accurately discriminate between participants who adhere to ART and those who do not. Nonetheless, younger age (21–30), achieving only elementary education and consuming alcohol had statistically significant associations with participants’ non-adherence to ART.

The clinical implication of missing one or more doses of ART are development of drug resistance and treatment failure [10, 17]. Although it has been documented that the best response to ART is seen when adherence is 100% [16], however, with the advent of new drugs such as the integrase strand transfer inhibitors and newer protease inhibitors, adherence with these drugs do not necessarily need to be 100% as the drugs could provide undetectable viral loads even with lower levels of adherence [38–40]. Nevertheless, the efficacy and durability of ART drug regimens still requires near perfect adherence rates as high as 95% or more [41–43].

While the level of ART non-adherence among participants in this study appears high (37.78%), it is within the range reported among participants in similar studies conducted elsewhere. We found no studies on ART non-adherence conducted in different regions of Cameroon prior this study, but the prevalence of ART non-adherence ranges from 7.5% in Lesotho [44] to over 39% in Australia [27]. In contrast, high ART adherence was also reported in other studies ranging from 62% in USA [45] to over 95% in Malawi [46].

A series of studies have documented the reasons for ART non-adherence including stigma, negative perception, lack of family and community support [47, 48]. Other factors such as disclosure of status, unemployment, lack of transport to go to the health facility for ART, insufficient feeding, inadequate follow ups, lack of patient confidentiality, lack of disability grants and alternative forms of therapy have also been reported as common obstacles to ART adherence [48]. Besides these, physical, economic and emotional stresses, travel from home, business with other things, depression, alcohol or drug use and ART dosing frequency have also been reported to hinder ART adherence [49, 50]. In this study however, some of the reasons for missing ART included forgetfulness, being busy with other things and travelling to a different location without drugs reported in 35.29%, 27.65% and 12.35% of participants respectively. However, only 54.67% of participants know ART should be taken life-long, 7.32% do not believe in the benefits of ART, 28.60% think taking ART gives an unwanted reminder of HIV status and 2.00% of participants said they have experienced health service discrimination while seeking ART services–these could be masked reasons for participants’ non-adherence to ART and reiterates a need to improve health workers attitudes towards patients, improve staff-patient-communication and proper counselling of patients on the importance of adhering to ART prior to their ART initiation.

Some studies have found that factors significantly associated with ART adherence include trust in medication, trust in healthcare system and interpersonal relationship with physicians and peers [45] and disclosure of HIV status, drug addiction, satisfaction with health service and perception of the need for medical treatment [51]. While our sample size was sufficient to estimate the proportion of ART non-adherence among participants, only a limited number of covariates appeared to have an association with non-adherence and could be considered as potential predictors—age, education, and alcohol consumption. Our findings are similar to those of other studies that found age [52, 53], education [19, 54, 55] and alcohol consumption [56] as factors associated with ART non-adherence. Nonetheless, our findings differ from studies that found ART non-adherence to be associated with side effects of the drugs, social isolation and complexity of the antiretroviral (ARV) regimens [57]; physical, economic and emotional stress [49]; poor or inability to follow prescribed treatment, no visits in a month, anxious and or depressed mood, difficulty in taking medication, cannabis consumption and receiving methadone treatment [58]; and being a female sex worker and suffering from moderate-to-severe depression [52].

### Limitations and strengths of this study

The level of ART non-adherence in this study could be influenced by population characteristics, the COVID-19 pandemic and the current political crisis in the country which might further be limiting access to ART. Although we sampled participants in three regions of the country, the sample may not have been representative of all PLWH and seeking ART services in these regions as the study did not include all treatment centres in each selected region. Furthermore, the study included only a cross section of PLWH in Cameroon. Thus, the prevalence of non-adherence obtained reflects only the detection of cases of non-adherence in our study settings and not necessarily the incidence of non-adherence or the overall prevalence of non-adherence in the population. Participants’ differences in access to HIV treatment may also contribute to an underestimation of the prevalence of non-adherence to treatment in patients more likely not to visit treatment centres for their treatment. Again, there is the possibility of response bias as participants may not have revealed the truth of their past adherence to treatment history. There may equally be recall bias as participants may not have fully remembered whether they have been non-adherent to HIV treatment or not.

Despite these limitations, the study was strengthened by the fact that it was conducted in multiple randomly selected settings reflecting the diversity of patients in the country, interviewers were trained to ensure appropriate data collection, and primary data that ensures that questions pertaining to this study are directly ascertained was collected. Our statistical analysis also allowed for a more accurate description of the prevalence of non-adherence and the factors more likely associated with non-adherence to HIV treatment in the context of Cameroon.

## Conclusion

A high proportion of participants are not adhering to their ART. However, some of the reasons for missing ART are forgetfulness, being busy with other things and travelling to another place without drugs. Some of the reasons for missing ART are masked in participants limited knowledge in taking their ART as prescribed, disbelief in the benefits of ART, feelings that taking ART gives an unwanted reminder of HIV status and experience of discrimination when using ART services. This underscores the need to improve the attitude of staff working in HIV treatment centres, improve staff-patient-communication and provide patients with appropriate counselling on the importance of adhering to their ART prior to their ART initiation. There was a significant relationship between non-adherence to ART and only three participants’ socio-demographic/ structural characteristics (younger age (21–30), attainment of only the primary education and alcohol consumption), other factors that could possibly contribute to participants’ non-adherence to ART needs to be explored. To guide policy making, future studies need to focus on assessing long-term ART non-adherence trends and determinants or predictors of ART non-adherence using larger samples of people living with HIV in many treatment centres and regions.

## Supporting information

S1 File Self-reported ART non-adherence questionnaire. (PDF)

S2 File Original datasets. (CSV)

## List of abbreviations

AIDS: Acquired Immune deficiency syndrome
aOR: Adjusted odds ratio
ART: Antiretroviral Therapy
ARV: Antiretroviral drugs
CD4: Cluster of differentiation 4
COVID-19: Coronavirus disease 2019
CI: Confidence interval
HIV: Human immunodeficiency virus
IQR: Interquartile range
IRB: Institutional Review Board
KM: Kilometers
N: Frequency
LMIC: Low and Middle-Income Countries
OR: Odds Ratio
PLWH: People living with HIV
PLWHA: People living with HIV/AIDS
Ref: Reference variable
SD: Standard deviation
SSA: Sub-Saharan Africa
USA: United States of America
XAF: African Financial Community Franc
