# Supplementary material for: Prevalence and factors associated with HIV treatment non-adherence among people living with HIV in three regions of Cameroon: A cross-sectional study
Source: PLoS One. 2023 Apr 4;18(4):e0283991. doi: 10.1371/journal.pone.0283991 (PMC10072448; doi:10.1371/journal.pone.0283991)
Supplement: S1 File — (PDF) [file pone.0283991.s001.pdf]

Self-reported ART non-adherence questionnaire

Dear respondent, thank you for accepting to participate in this study. The following statements are some things people say about their ART experience. Please listen attentively, keeping in mind the HIV treatment you are receiving; (We would like to know how you feel about the ART you are taking. Your answers to the questions will help us understand what works and what doesn't, and how we can develop strategies that will improve patients' adherence to ART). We are very interested in your candid opinion on your experience taking ART.

A) Socio-demographic/structural characteristics

i) Region of residence: 0=North West…., 1=South West…, 3=Littoral….
ii) Your Age………………………years
iii) Sex (please tick): 0 = Male..., 1 = Female………
iv) Marital status (select one): 0=Single…,1=Married…, 2=Devorced…, 3=Separated…,4=Never married…
v) Level of education (select one): 0=Primary…, 1=Secondary…, 2=High school…, 3=University…
vi) Religion: 0=Muslim…, 1=Catholic…, 2=Protestant…, 3=Pentecostal…, 4=animist….
vii) Working status (select one): 0=no job…., 2=self-employed…, 3=government staff…, 4=private staff…
viii) What is your estimated monthly income? …………………………………
ix) What is the distance from your house to this Centre? ……. Kilometers
x) How long does it take you to get to this centre? ….. Minutes
xi) How do you get here? 0=by foot…, 2=by bike…, 3=by car/taxi…
xii) How many people are living with you in the same house?......
xiii) Who are the persons living together with you? (select all that apply) 0=spouse/partner…, 1=children…, 2=parents…, 3=relatives/friend…, 4=Nobody...
xiv) Have you disclosed your HIV status to family or friend? 0=No…., 1=Yes…

B) Health risk behaviours

a. Are you currently smoking? 0=No…., 1=Yes….
b. Do you drink alcohol? 0=No…, 1=Yes…
c. Do you use drugs that are not prescribed for you? 0=No…, 1=Yes…
d. Do you have multiple sex partners (more than one at a time)? 0=N0…, 1=Yes…
e. Do you carryout physical exercises? 0=No..., 1=Yes…

C) Medical history and ART adherence experience

1. Have you ever been admitted to hospital for HIV related issues? 0=No…, 1=Yes…
2. If yes, what were the reasons for hospital admittance? (tick all that apply): 0=chronic diarrhea…, 1=chronic herpes simplex…, 2=TB/pneumocystis…, 3=meningitis…, prolong fever…, 4=weight loss…, 5=Jaundice…, 6=other (specify)………………
3. How long has it been since you were first diagnosed HIV positive?......Months
4. How long have you been taking ART? .... Months
5. What is your ART dosing frequency per day? ……….. times/day
6. How is your health status since ART initiation? (Select one): 0=Worse…, 1=stable…, 2=better…
7. Have you ever missed taking your ART in the last month? 0=No …, 1=Yes …
8. If yes to question 7, how many times have you missed taking ART in the past 4 weeks? ……………..
9. What was the reason(s) for missing to take your ART (tick all that apply): 0=Forgot …, 1=Busy …, 2=Too many pills/taste of medicine …, 3=Long distance to hospital …, 4=Run out of medicine …, 5=Severe symptoms/difficulty to follow up …, 6=Stigma …, 7=Other, specify ……………………………………………………
10. Have you ever had any side effects of ARV? 0=No…, 1=Yes…
11. Have you ever missed an appointment for ART services? 0=No …, 1=Yes …
12. Please indicate how strongly you AGREE or DISAGREE with each of the following statements. (Tick only one, where appropriate).

		Strongly agree	Agree	Uncertain	Disagree	Strongly disagree
a	I know that ART is lifelong					
b	I do not believe in the benefits of ART					
c	Taking tablets gives me an unwanted reminder that I have HIV					
d	I am worried that in the future, my medication will stop working for me					
e	I have experienced physical adverse events in the last 12 months					
f	I have experienced health service discrimination in the last 2 years					

Source: adapted from (Glass *et al.*, 2006; Duff *et al.*, 2010; Grierson *et al.*, 2011; Hansana *et al.*, 2013; Chaiyachati *et al.*, 2014)**.**
